# Skull Vibration Induced Nystagmus Test: Correlations with Semicircular Canal and Otolith Asymmetries

**DOI:** 10.3390/audiolres11040056

**Published:** 2021-11-15

**Authors:** Christol Fabre, Haoyue Tan, Georges Dumas, Ludovic Giraud, Philippe Perrin, Sébastien Schmerber

**Affiliations:** 1 Department of Oto-Rhino-Laryngology, Head and Neck Surgery, University Hospital, 801321 Grenoble, France; georges.dumas10@outlook.fr (G.D.); Lgiraud2@Chu-Grenoble.Fr (L.G.); sschmerber@chu-grenoble.fr (S.S.); 2 Department of Otolaryngology H & N Surgery, Shanghai Ninth People’s Hospital, Shanghai Jiaotong University School of Medicine, Shanghai 200011, China; thycxf@126.com; 3 EA 3450 DevAH, Development, Adaptation and Handicap, Faculty of Medicine and UFR STAPS, University of Lorraine, 54578 Villers-lès-Nancy, France; philippe.perrin@univ-lorraine.fr; 4 Department of Paediatric Oto-Rhino-Laryngology, University Hospital of Nancy, 54500 Vandoeuvre-lès-Nancy, France; 5 BrainTech Lab., INSERM UMR 1205, 38000 Grenoble, France

**Keywords:** skull vibration induced nystagmus, dizziness, unilateral vestibular loss, vestibular functional tests

## Abstract

Background: To establish in patients with peripheral vestibular disorders relations between skull vibration-induced nystagmus (SVIN) different components (horizontal, vertical, torsional) and the results of different structurally related vestibular tests. Methods: SVIN test, canal vestibular test (CVT: caloric test + video head impulse test: VHIT), otolithic vestibular test (OVT: ocular vestibular evoked myogenic potential oVEMP + cervical vestibular evoked myogenic potential cVEMP) performed on the same day in 52 patients with peripheral vestibular diseases (age < 65 years), and 11 control patients were analyzed. Mixed effects logistic regression analysis was performed to assert whether the presence of nystagmus in SVIN (3D analysis) have an association with the presence of peripheral vestibular dysfunction measured by vestibular explorations (CVT or OVT). Results: We obtained different groups: Group-Co (control group), Group-VNT (dizzy patients with no vestibular tests alterations), Group-O (OVT alterations only), Group-C (CVT alterations only), Group-M (mixed alterations). SVIN-SPV horizontal component was significantly higher in Group-M than in the other groups (*p* = 0.005) and correlated with alterations of lateral-VHIT (*p* < 0.001), caloric test (*p* = 0.002) and oVEMP (*p* = 0.006). SVIN-SPV vertical component was correlated with the anterior-VHIT and oVEMP alterations (*p* = 0.007; *p* = 0.017, respectively). SVIN-SPV torsional component was correlated with the anterior-VHIT positivity (*p* = 0.017). SVIN was the only positive test for 10% of patients (83% of Group-VNT). Conclusion: SVIN-SPV analysis in dizzy patients shows significant correlation to both CVT and OVT. SVIN horizontal component is mainly relevant to both vestibular tests exploring lateral canal and utricle responses. SVIN-SPV is significantly higher in patients with combined canal and otolith lesions. In some patients with dizziness, SVIN may be the only positive test.

## 1. Introduction

**Figure 1:**
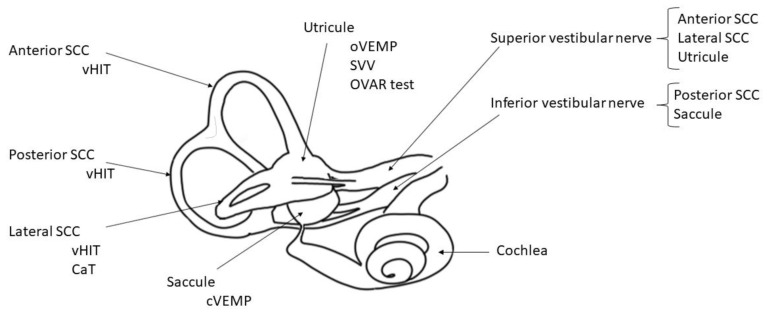
Inner right ear diagram with vestibular structures and their respective tests. CaT: caloric test; OVAR test: off vertical axis rotation test; SCC: semi-circular canal; SVV: subjective visual vertical; VHIT: video head impulse test; VEMPs: vestibular evoked myogenic potential (ocular and cervical).

Vestibular disorders performant analysis is currently based on multifrequency and topographic tests which selectively explore all the different vestibular structures (Figure 1).

Skull vibration induced nystagmus test (SVINT) is a non-invasive, well tolerated test which complements other vestibular tests in the vestibule multifrequency analysis [1]. Performed with a hand held 100 Hz vibrator, the skull vibration induced nystagmus (SVIN) is reproducible on both mastoids, presents no after nystagmus (no nystagmus reversal inversion) and is not modified by vestibular compensation [2,3]. SVIN direction can be horizontal, vertical, or torsional, suggesting that SVIN is a global vestibular response [1,2,4]. SVIN is essentially produced by the response on the healthy side and acts as a vestibular Weber test, the induced nystagmus shows quick phases beating usually toward the side opposite to the lesion in unilateral vestibular-loss [5,6]. This test allows a simple access to the vestibulo-ocular reflex (VOR) very high frequencies which are not analyzed by common vestibular tests which address low or middle range frequencies [2] and has been described in numerous vestibular pathologies [1,5,6,7,8].

Bone conducted vibrations stimulate both canal and otolith structures [9,10] but SVIN origin is not clearly determined: the only horizontal canal for its horizontal component [11], other canals for its torsional and vertical components [12,13,14], otolith structures, both [15] or cervical proprioceptive stimulation [7,16] were proposed.

A two-dimensional (2D) study from Zhang et al. has recently showed a strong relationship between SVINT results and tests measuring SCC asymmetry but a less important correlation with otolith function [17]. Fugimoto et al. tested patients with UVL (vestibular neuritis (VN), Menière’s disease (MD), unilateral vestibulopathy and vestibular migraine under videoscopy and showed that VIN was associated with dysfunction in all SCC and utricle [18].

We hypothesize so a main contribution of canals to SVIN and a possible modulation of responses by otolith afferents.

This study aimed to assess whether the different components (horizontal, vertical, torsional) of SVIN at 100 Hz can be correlated with canal vestibular tests (CVT) and otolith vestibular tests (OVT) and their corresponding vestibular structures to precise SVIN origin and its application in clinical routine. We performed a 3D analysis of the SVIN components to establish with more accuracy possible correlations with vestibular structures.

## 2. Materials and Methods

### 2.1. Terminology

Nystagmus axis: the axis can be horizontal, vertical, or torsional. Nystagmus direction can be right or left, upward or downward, or rotatory clockwise or counterclockwise.

### 2.2. Participants

In this monocentric prospective study, 52 patients of age < 65 years old were included with different peripheral vestibular pathologies: 18 had a vestibular neuritis, 8 a Menière’s disease, 11 a recurrent vestibulopathy and 15 had intratympanic gentamicin (ITG). The inclusions were performed consecutively within a 2-month period. Different types of partial peripheral unilateral vestibulopathies (PUVL) were accepted (intentionally included) to assess possible different topographic vestibular lesions. The participants gave their written informed consent to contribute to this study in accordance with the Helsinki II declaration.

MD Inclusion criteria corresponded to those defined by Lopez-Escamez [19]. Vestibular neuritis responded to the criteria edict of 2015 [20], and recurrent vestibulopathy BRV (or benign recurrent vestibulopathy) to those defined by Slater and Van Esch [21,22] and more recently specified by Ducroz [23] and Dlugaiczyk [24] (migraineurs were excluded and only patients corresponding to the definition of recurrent vestibular symptoms not otherwise specified, i.e., RVS NOS were included).

Exclusion criteria were: patient’s age > 65 years (due to possible modifications of vestibular explorations related to aging as for VEMP [25]) and <18 years old; refusal of patients to enter the study. MRI was performed when necessary to establish positive or differential diagnosis in 42 cases.

Patients with neurological pathologies, spontaneous nystagmus or post-surgical total UVL were excluded as patients with psychiatric disorders or under strong sedative medications.

Mean age was 57years old (18–65), and gender displayed 29 males and 23 females. All cases were documented by clinical examination, caloric test (CaT), cervical vestibular evoked myogenic potential (cVEMP), ocular vestibular evoked myogenic potential (oVEMP), video head impulse testing (VHIT) and SVINT. CaT and VHIT are canal vestibular tests (CVT). cVEMP and oVEMP are otolith vestibular tests (OVT).

The subjective visual vertical (SVV) exploration was not performed due to the possible influence of vestibular compensation which may normalize the test in time.

Eleven healthy subjects were recruited as controls: mean age 55 years old (24–65 years), 4 males and 7 females. Their healthy status was defined by the absence of cochleo-vestibular symptoms or history and normal common vestibular tests and hearing [4].

All the subjects gave their informed consent before inclusion in the study. All the experiments were performed in accordance with the Helsinki II Declaration.

### 2.3. Vestibular Testing Methods

The canal function and particularly the horizontal SCC function have been studied at different frequencies with conventional tests: low frequencies for the caloric test and high frequencies with the VHIT since results may be different following frequencies in a few pathologies such as Menière’s disease.

- SVINT: The vibratory stimulation was applied in all participants during 10 s successively to the right and left mastoid processes using the hand-held V.VIB 3F stimulator (Synapsys, Marseille, France) at a stimulus frequency of 100 Hz. A 3D videonystagmographic (VNG) device (Synapsys, Marseille, France) was used to detect the SVIN occurrence and to measure its slow-phase eye velocity for horizontal (H-SPV, °/s), vertical (V-SPV, °/s) and torsional components (T-SPV, °/s). A positive SVINT is defined by consistent responses on both mastoids and with a H-SPV > 2°/s [1]. The 3D VNG device from synapsis has proven its efficiency and accuracy by measuring and an objective torsional vertical component in posterior canal benign paroxysmal positional vertigo (BPPV) and only horizontal component in horizontal SCC BPPV [26].
- VHIT: The participants were installed in front of a camera device VHIT Ulmer II 0 14 (Synapsys, Marseille, France). Semicircular canals (SCC) were tested in pairs according to the planes of stimulation (horizontal plane, right–anterior–left–posterior plane, left–anterior–right–posterior plane). Ten valid head-impulses were needed for each canal. The gains were given for: L-VHIT (lateral SCCs), A-VHIT (anterior SCCs) and P-VHIT (posterior SCCs). A gain < 0.8 for L-VHIT and <0.7 for A-VHIT and P-VHIT and asymmetry between canals >20% were considered abnormal.
- VEMP test: Recordings were performed with Neuro-Audio System (Russia). The VEMPs were performed with the Neuro-Audio device (Neuro Audio Collin Medical France). The stimuli delivered with headphones (TDH 39) were short-tone bursts at a frequency of 500 Hz and intensities ranged from 110 to 70 dB. The stimulus was a 500 Hz air-conducted tone-burst (rise/fall time 1 ms, plateau 2 ms; ear headphones TDH39). For oVEMP, the active electrode was positioned approximately 1 cm below the lower eyelids, the ground electrode on the high forehead (Fz) and reference electrode was placed on the chin. The subjects were instructed to direct their gaze at a visual target with a vertical elevation of about 30 degrees. For cVEMP, the ground electrode was placed on the medium part of the forehead (Fz), the active electrodes at the junction of the superior one third and inferior two-third of both sternocleidomastoid muscles, the reference electrode was located over the upper sternum. The subjects were informed to turn the head in the opposite direction to the stimulated side. A difference in amplitude between both sides higher than 25% was considered to be significant to show an asymmetry (positive test).
- CaT: The bithermal caloric test protocol (30 °C, 44 °C) was performed on each ear with VNG device (Synapsys, Marseille, France) following the Fitzgerald-Hallpike technique. A unilateral vestibular weakness was considered significant when the difference between both ears was greater than 20% [27]. The percentage of hypofunction (CaT) and relative preponderance (% RP) were used for the quantitative study and correlations with SVIN-SPV.

### 2.4. Procedure

The five tests were performed on the same day and by the same team in the following sequence: SVINT, VHIT, cVEMP, oVEMP and CaT with a delay of half an hour between each test. A qualitative analysis was performed by evaluating groups and their correlation with SVIN positivity with Fisher tests and in a multivariate logistic regression model. A quantitative evaluation was also performed by correlating the asymmetry of gains in percentage of VHIT, the caloric hypofunction in percentage, relative preponderance, the oVEMP and cVEMP asymmetry rate in percentage with the SVIN-SPV.

### 2.5. Statistical Analysis

R v3.5.1 software was used for statistical analysis with MASS and leaps packages. Data are presented as mean ± standard deviation (SD), unless otherwise specified. Parametric or non-parametric tests were used according to distribution of variables. Chi2 and Fisher tests were used for positivity of test repartition.

For the relation of SPV and quantitative gains asymmetries, a fitted multiple linear regression model was used. The predictive variables for horizontal vertical or torsional SPV were results of oVEMP, cVEMP, L-VHIT, A-VHIT, P-VHIT, CaT, and RP. Only observations of Groups O, C and M were integrated. We used a backward selection procedure of the candidate variables based on the Akaike’s information criteria (AIC). The normal and homoscedastic distribution of residuals was verified. Because of multiple outliers, we then applied a robust linear model on selected variables to ensure regression coefficients. A threshold *p*-value < 0.05 was considered significant.

The statistical analysis was approved by the Department of Statistics of our University Medical Center.

## 3. Results

### 3.1. Identified Groups of Patients

The population was divided into five groups: Group-Co (control group without vestibular symptoms and normal CVT and OVT), Group-VNT (vestibular symptoms, with normal OVT and CVT for both CaT and VHIT), Group-O (otolithic group, OVT alterations and normal CVT), Group-C (canal group, CVT alterations and normal OVT), and Group-M (mixed group, with combined CVT and OVT alterations). CVT alterations meaning that at least one canal result was altered; OVT alterations means that at least one otolithic test was modified. Table 1 reports distribution of diagnosis in groups (*p* < 0.01).

In the control group (Co), 2 control subjects had a low intensity SVIN (<2°/s) not reproducible on each mastoid or changing of direction and were consequently considered negative. Other subjects had no SVIN.

**Figure 2:**
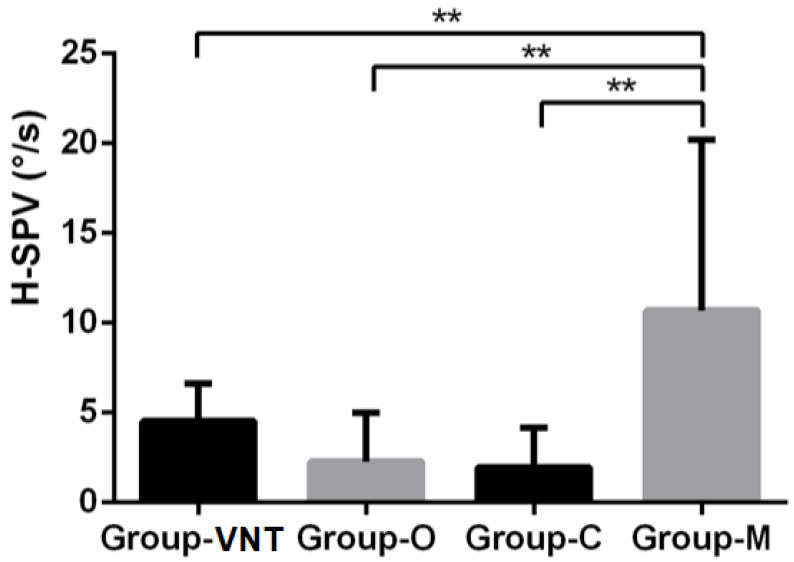
H-SPV bar chart with standard error by pathological groups. (**: *p* < 0.01).

SVIN positivity repartition was not different in the different pathological groups (Fisher exact test: *p* = 1). However, the value of SVIN-SPV horizontal component measured at 10.7 ± 9.50°/s in Group-M was significantly higher than in the other groups (Kruskal–Wallis test: *p* = 0.0055). There were no differences among the other groups (Table 2 and Figure 2).

The comparison of caloric hypofunction and L-VHIT asymmetry between Group-C and Group-M did not reveal statistical difference (*p* = 0.97 and *p* = 0.35, respectively).

In Group-VNT, 83% of patients had a positive SVIN, meanwhile all the other tests were negative. These patients represent 11% of the all population analyzed (Groups-N, O, C, M) (i.e., SVIN is the only positive routine test for approximately 10% of patients with vertigo). Conversely, SVIN is negative in 13% of patients with positive CVT and OVT.

### 3.2. SVIN Horizontal Component

**Figure 3:**
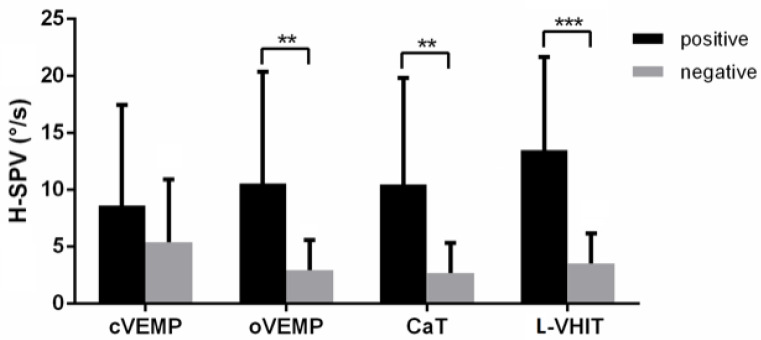
H-SPV bar chart with standard error comparison between positive and negative results of vestibular tests. (**: *p* < 0.01, ***: *p* < 0.001).

The horizontal component SVIN-SPV was analyzed and compared to results in each other vestibular test. SVIN positivity was significantly different in face of positive/negative CaT (*p* < 0.05), positive/negative oVEMP (*p* < 0.05) but not in front of modalities of L-VHIT (*p* = 0.13) and cVEMP (*p* = 0.29) (Table 3). However, there was a significant difference for H-SVIN-SPV values regarding L-VHIT results (*p* < 0.001). Figure 3 shows that there was no difference in H-SPV with positivity of cVEMP (*p* = 0.581), but positive groups of oVEMP, CaT and L-VHIT were significantly different from their respective negative groups (*p* = 0.006, 0.002, <0.001 respectively) (Table 3). The introduction of these 3 variables in the logistic regression models showed an odd ratio for oVEMP: 2.06 (+/−0.57, *p* = 0.024), for L-VHIT: 3.13 (+/−1.5, *p* = 0.03) and for CaT: 4.34 (+/−4.5, *p* = 0.1).

For patients in Group-M with positive CaT and L-VHIT: the SVIN-SPV was 17.06 ± 8.48°/s when oVEMP and cVEMP were both positive; 16.73 ± 9.89°/s when oVEMP was solely positive (*p* = 0.85) and 6.5 ± 4.79°/s when cVEMP was solely positive (*p* < 0.001).

The backward selection of variables procedure found the best linear model with the smallest AIC:

H-SPV ~ oVEMP + L-VHIT + CaT (*p* < 0.001, AIC = 140). (1)

The results of the robust linear multiple regression indicated the three predictors explained 52% of the variance (R² = 0.518, freedom degrees = 65, M-estimator statistics = 6.059). VHIT horizontal canal asymmetry was significantly correlated with the horizontal SVIN-SPV (b = 39, bSE = 4.90, *p* < 0.001), idem for oVEMP (b = 0.11, bSE = 0.03, *p* < 0.001) and for CaT (b = 0.1, bSE = 0.05, *p* = 0.04).

### 3.3. SVIN Vertical and Torsional Components

**Figure 4:**
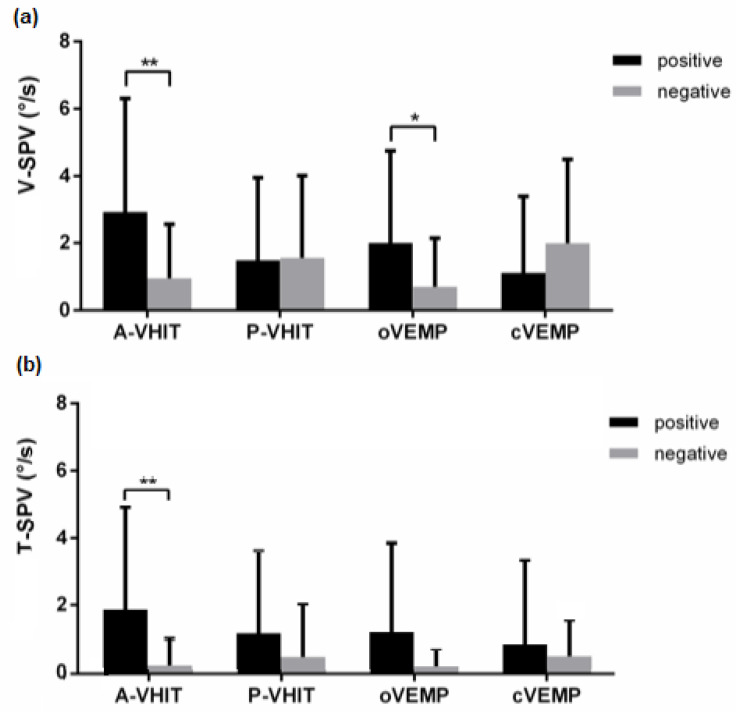
V-SPV (**a**) and T-SPV (**b**) bar charts with standard error comparison between positive and negative results of vestibular tests. (*: *p* < 0.05, **: *p* < 0.01).

SVIN vertical and torsional components showed lower SPV. For each component the SPV mean value was 1.52 ± 2.41°/s and 0.72 ± 1.93°/s, respectively. The A-VHIT and oVEMP positivities were correlated with an increased SVIN vertical component SPV (*p* = 0.007, 0.017, respectively). The V-SPV was not correlated with the positivity or negativity of P-VHIT and cVEMP results (Figure 4). In multivariate logistic regression, odd ratios were 2.87 +/−1.01 (*p* = 0.041) for A-VHIT and 4.14 +/−2.7 (*p* = 0.03) for oVEMP. A-VHIT and o-VEMP results were designed as predictors for V-SVIN-SPV values (*p* = 0.002, AIC = 182) when using multiple regression linear model selected by AIC but the value of the A-VHIT coefficient was not statistically different of 0 (*p* = 0.19). Idem, the robust regression returned a significant coefficient for oVEMP as predictor (b = 0.060 bSE = 0.02 *p* = 0.0354) but not for A-VHIT (b = 5.8 bSE = 7.42 *p* = 0.43). A-VHIT and oVEMP explained 33% of the V-SPV variance.

For the SVIN-SPV torsional component (T-SPV): only the A-VHIT positivity was significantly related to higher values of the T-SPV (odd ratio: 1.82+/− 0.75, *p* = 0.017). Conversely, T-SPV values do not change with P-VHIT, oVEMP, cVEMP positivities (*p* = 0.215, 0.162 and 0.581, respectively) (Figure 4).

The variable selected in multiple regression were oVEMP, A-VHIT and CaT (*p* < 0.001, AIC = 171) which explained 44% of the T-SPV variance, but robust regression did not permit to ensure the CaT effect (*p* = 0.45) contrary to oVEMP (b = 0.035, bSE = 0.01, *p* = 0.002) and A-VHIT (b = 9.86, bSE = 3.01, *p* = 0.0048).

Among this population, two definite inferior vestibular neuritis with positive cVEMP and P-VHIT, but normal CaT oVEMP, A-VHIT and L-VHIT, had no SVIN.

## 4. Discussion

This is the first study to perform a 3D analysis of SVIN to explore a possible correlation with different vestibular structures. This study aimed to assess whether the different components (horizontal, vertical, torsional) of SVIN at 100 Hz can be correlated with canal vestibular tests (CVT) and/or otolith vestibular tests (OVT) and their corresponding vestibular structures to orientate toward a probable origin of SVIN and its application in clinical routine. This is the first study to perform a correlation of the SVIN 3D components with other vestibular tests.

Although it is clearly known that BCV stimulate equally otolith and canal structures at 100 Hz the respective contribution and proportion of these structures in the production of the induced nystagmus and SVIN origin is not clearly determined. Fluid displacement generated by BCV, or air-conducted sound deflects the hair bundle and activates different receptors: Curthoys reported that BCV activated canal, utricular and saccular irregular neurons in intact guineapigs at 100 Hz [10]. Our results showed that, at this frequency, equal SVIN-SPV was observed in patients with either CVT or OVT positive, but that SVIN-SPV was significantly higher in patients with both CVT and OVT altogether positive (Group-M) suggesting that the contribution to SVIN is relevant from both canal and otolith stimulation with a synergistic effect.

There was no difference in terms of horizontal canal asymmetry between Group-M and Group-C, so a more important damage to horizontal SCC in Group-M cannot be the single argument to explain this difference for SVIN-SPV.

Some authors hypothesized a less important utricular contribution involving converging neurons in the vestibular nucleus for the horizontal component to modulate canal responses [14,28].

Dumas et al. suggested in UVL patients [12] and Dlugaiczyk et al. [13] concluded in animals that horizontal SCC contribution is dominant over otolith in the constitution of SVIN at 100 Hz since in these cases the horizontal component is mostly represented; otherwise in superior semi-circular canal dehiscence, superior SCC contribution is represented by vertical and torsional components [12].

Zhang et al. showed in a 2D analysis of SVIN that there was a fair correlation between SVIN horizontal component and SCC function modifications, but that these correlations were less important for otolithic explorations as observed in our study [17]. Fugimoto et al. concluded SVIN was associated with abnormal function in all SCC and utricle [18].

In a recent article concerning lateral canal occlusion in a human model of patients with MD, a significant correlation between CVT alterations and SVIN horizontal component has been showed [29].

The vertical and torsional components SVIN-SPV are small. A significant correlation between anterior SCCs results (A-VHIT) and SVIN suggests strongly that the anterior SCCs stimulation by skull vibration contributes to the SVIN vertical and torsional components. A similar correlation has been observed for oVEMP. Suzuki in 1969 [30] demonstrated in cat that utricular nerve stimulations induce also slow eye vertical movement. In our series, no correlation has been observed with tests exploring the posterior SCCs (P-VHIT).

The saccular responses are mainly represented at the cervical level (sacculo colic reflex explored in clinic by cVEMP) but poorly on extra ocular muscles (VOR) although sacculo ocular connection is signaled by Isu and Goto in cats to induce vertical eye movements [31,32].

These results suggest that SVINT explores mainly horizontal SCC, superior SCC and utricle corresponding to vestibular structures driven by the VIII th nerve’s superior branch. Structures innerved by the inferior vestibular nerve are poorly or not represented, as suggested by the two cases of inferior vestibular neuritis with no responses induced by SVINT.

In some vertiginous patients who presented negative results to other routine classic vestibular tests (Group VNT) SVIN was the only positive test. We hypothesize that SVIN positivity corresponds to a primarily canal response at high frequencies not explored by caloric test (low frequencies) or VHIT (middle range frequencies). We assume that SVIN-test complements the multi-frequency vestibular evaluation particularly in certain cases of BRV [23].

One important limitation of our work is the small number of patients in each subgroup explained by scarcity of pure vestibular lesions. Conversely, the use of robust linear regression models has permitted to reduce outlier effects. Furthermore, all vestibular explorations were performed by the same examiner and on the same day to avoid bias related to force variation application and to vestibular fluctuations due to pathology as in MD. This study constitutes the first 3D analysis for such correlations

## 5. Conclusions

A significant correlation was observed between SVIN and vestibular tests exploring the lateral SCC, anterior SCC and utricle corresponding to the superior VIII th nerve root territory. Our results suggest strongly that these structures contribute for a large part to SVIN different components. SVIN explores globally otolith and canal structures and complements other vestibular tests at high frequencies. However, the SVIN horizontal dominant component in UVL at 100 Hz suggests a main horizontal canal contribution and accessorily of the utricle. SVIN may be also the only positive vestibular test in some dizzy patients.

## Figures and Tables

**Table 1:** Number of diagnoses by group.

Pathology	Group-VNT	Group-O	Group-C	Group-M
VN	1	0	2	15
MD	1	2	3	2
BRV	4	2	1	4
ITG	0	2	1	12

VN = vestibular neuritis; MD = Meniere’s disease; BRV = recurrent vestibulopathy; ITG = intratympanic gentamycin injections; Group-VNT = no alteration of CaT, VHIT, oVEMP, cVEMP; Group-O = isolated otolith alterations (oVEMP and/or cVEMP); Group-C = isolated canal alterations (CaT and/or VHIT); Group-M = both otolith and canal alterations.

**Table 2:** SVIN presence and horizontal SPV value in the different groups N, C, O, M.

	Group-VNT	Group-O	Group-C	Group-M	*p*-value
Cases (n)	6	6	6	34	
SVIN (n)	5	5	5	30	*p* = 1
SVIN (%)	83	83	83	88	
H-SPV (°/s)	4.52 ± 2.12	2.29 ± 2.72	1.96 ± 2.21	10.7 ± 9.50	*p* < 0.01
95% CI	1.73	2.22	1.80	3.26	

n = number; SVIN (n) = number of positive SVIN; SVIN (%) = percentage of positive SVIN (>2°/s). H-SPV (°/s) = Horizontal slow phase velocity of SVIN (mean ± SD). Group-VNT = no alteration of CaT, VHIT, oVEMP, cVEMP; Group-O = isolated otolith alterations (oVEMP and/or cVEMP); Group-C = isolated canal alterations (CaT and/or VHIT); Group-M = both otolith and canal alterations.

**Table 3:** SVIN presence and horizontal SPV value by result of different vestibular tests.

	CaT			L-VHIT			oVEMP			cVEMP		
	+	−	*p*-Value	+	−	*p*-Value	+	−	*p*-Value	+	−	*p*-Value
Cases (n)	27	25		17	35		38	14		33	19	
SVINT (n)	25	17	0.036	16	26	0.13	34	8	0.016	25	17	0.29
SVINT+ (%)	93%	68%		94%	74%		89%	57%		76%	89%	
SVIN-SPV (°/s)	10.5 ± 8.9	2.7 ± 2.8	<0.01	14.1 ± 7.3	2.6 ± 3.5	<0.001	10.6 ± 10.6	3.0 ± 2.3	<0.01	8.6 ± 8.6	5.4 ± 5.9	0.58
95% CI	3.46	1.13		3.56	1.20		3.45	1.24		3.02	2.76	

n = number of cases; SVIN (n) = number of positive SVIN; SVIN+ (%) = percentage of positive SVIN; H-SPV (°/s) = horizontal slow phase velocity in degree per second (mean ± SD); + = positive results (asymmetry); − = negative results (no difference between sides).
